# Supplementary figures and images for: Sustained store-operated calcium entry utilizing activated chromatin state leads to instability in iTregs
Source: eLife. 2023 Dec 6;12:RP88874. doi: 10.7554/eLife.88874 (PMC10699804; doi:10.7554/eLife.88874)

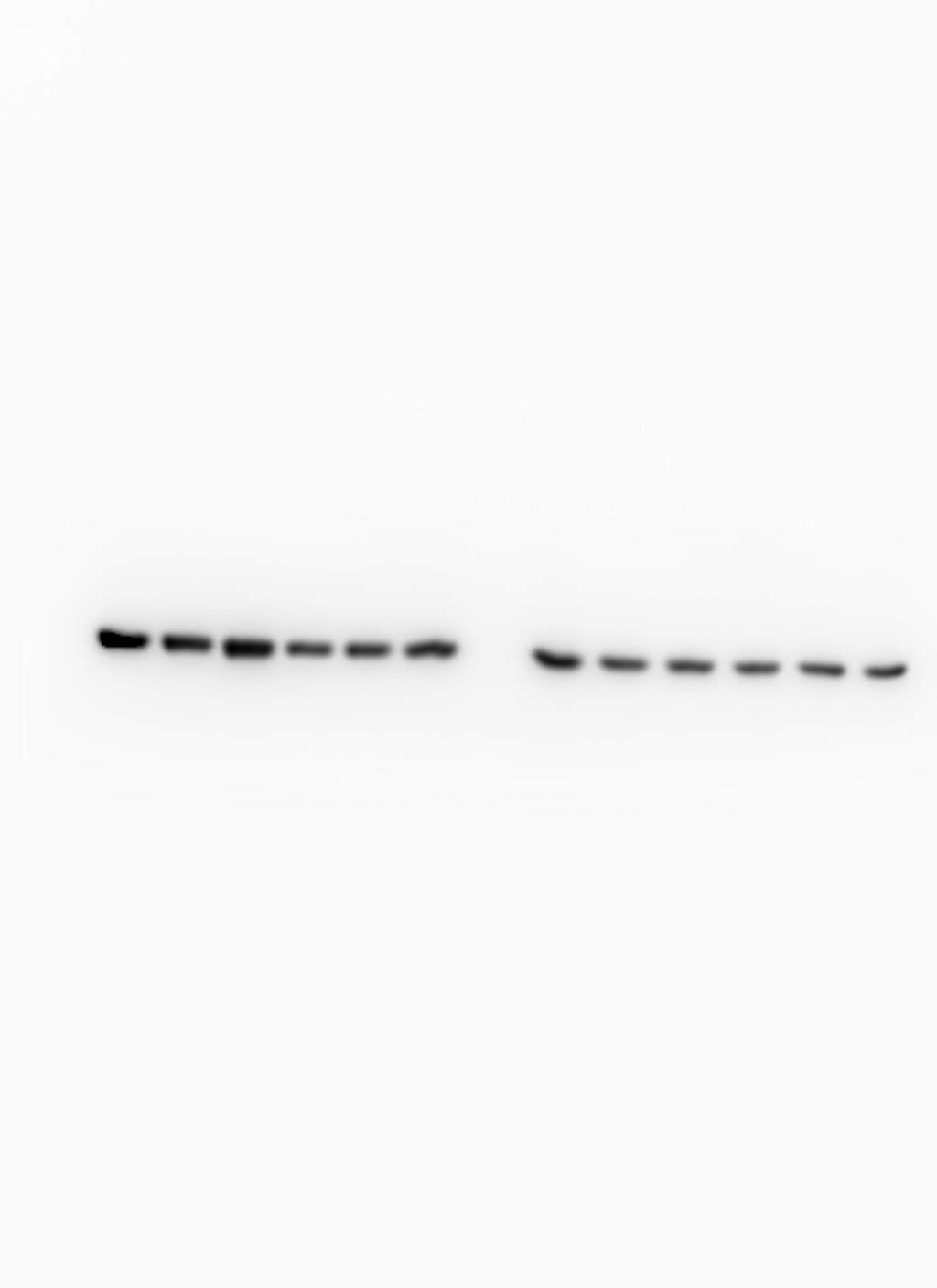

Supplement: Figure 2—source data 3. [file elife-88874-fig2-data3.zip › Fig 2D/Tconv&tTreg Actin.tif]

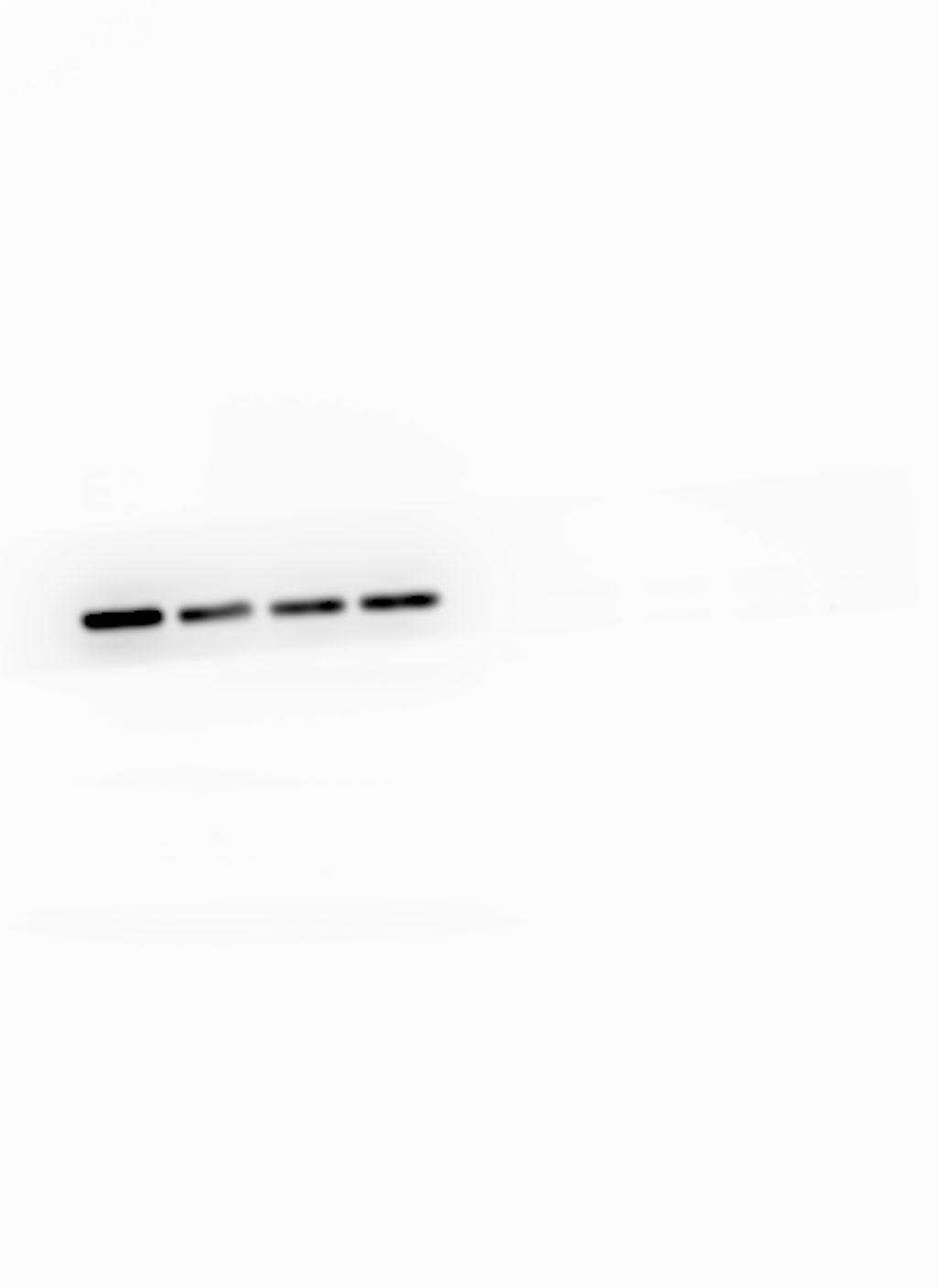

Supplement: Figure 2—source data 3. [file elife-88874-fig2-data3.zip › Fig 2D/iTreg Gapdh.tif]

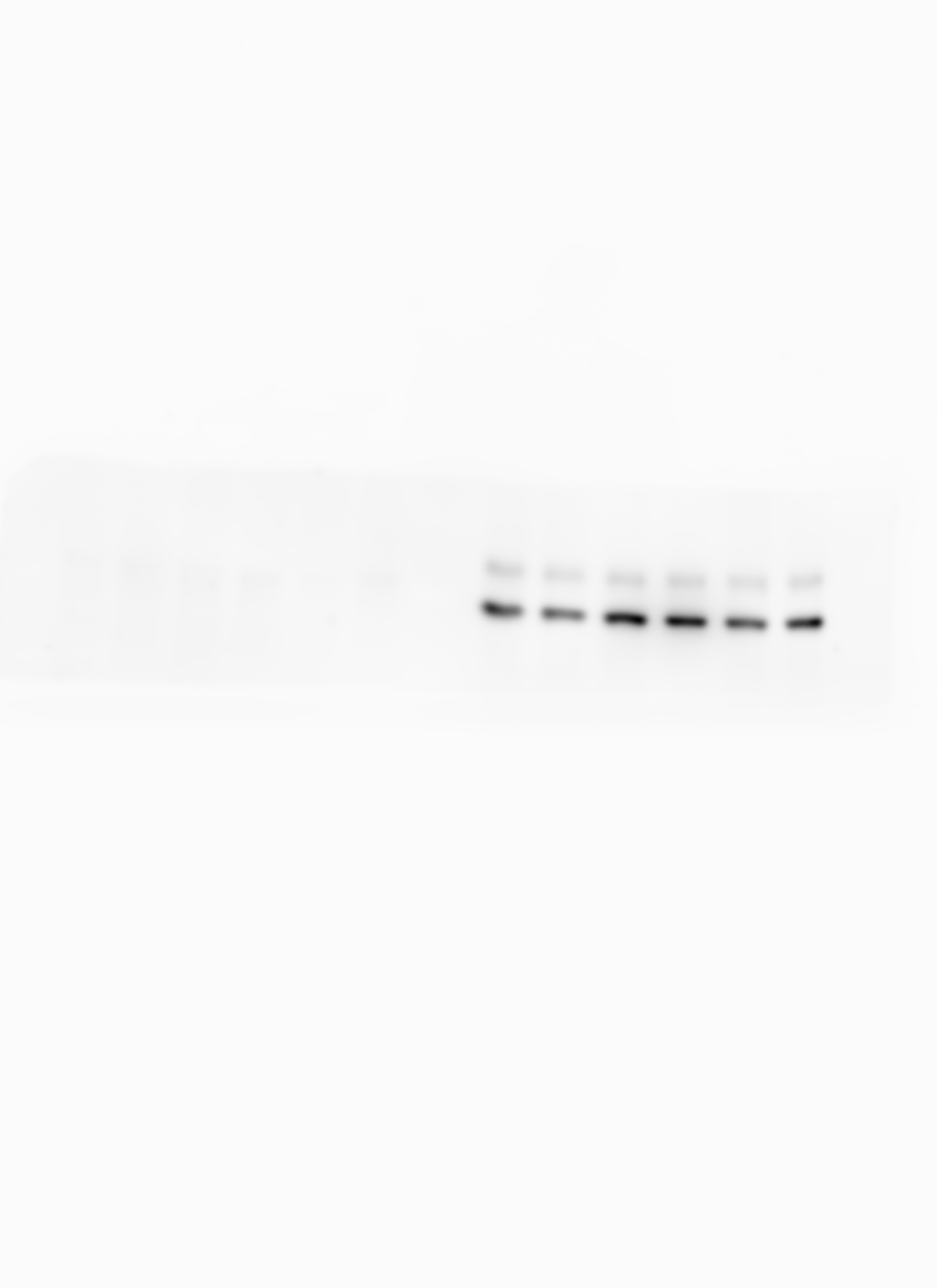

Supplement: Figure 2—source data 3. [file elife-88874-fig2-data3.zip › Fig 2D/Tconv&tTreg Lamin A:C.tif]

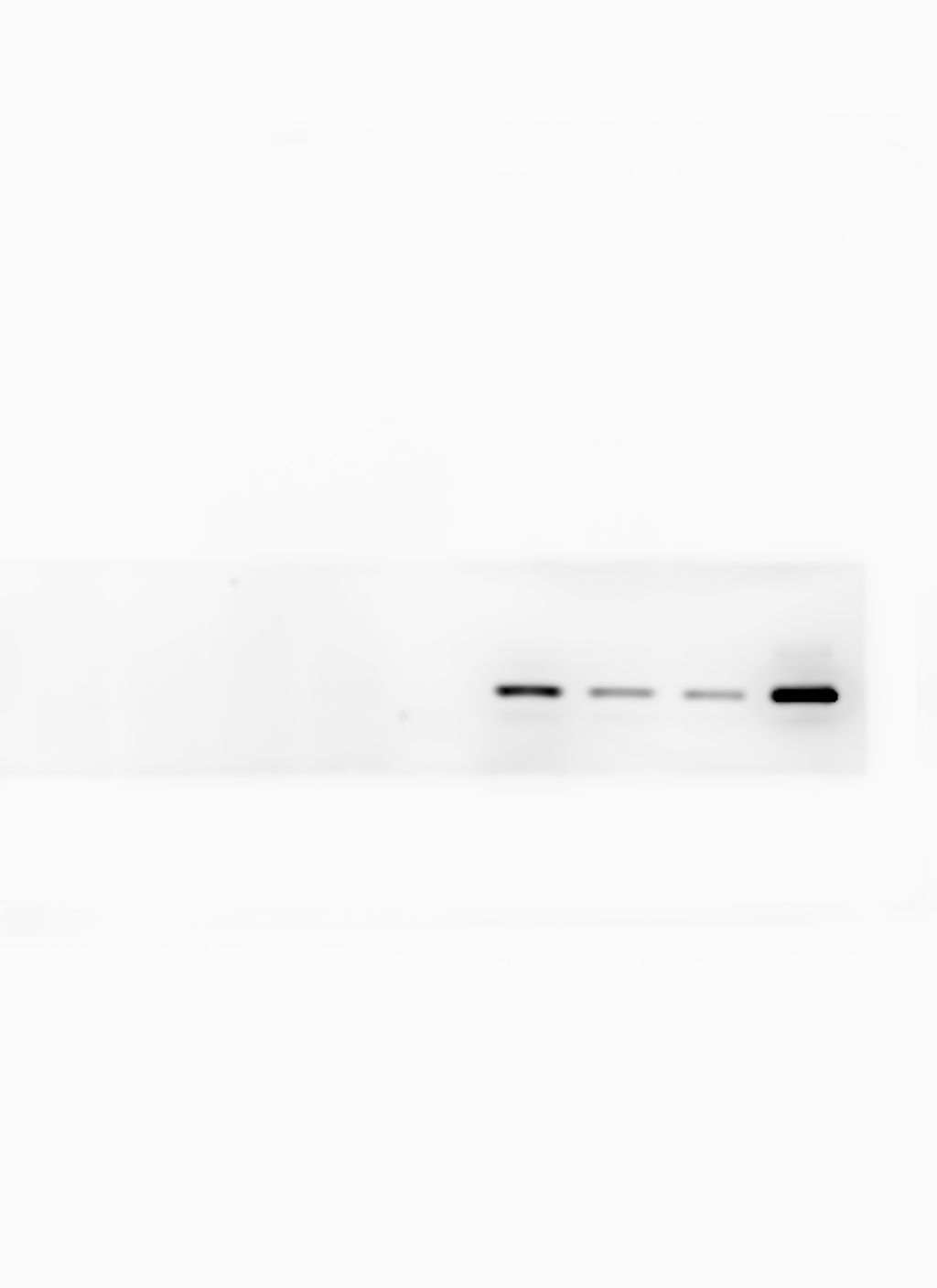

Supplement: Figure 2—source data 3. [file elife-88874-fig2-data3.zip › Fig 2D/iTreg Lamin A:C.tif]

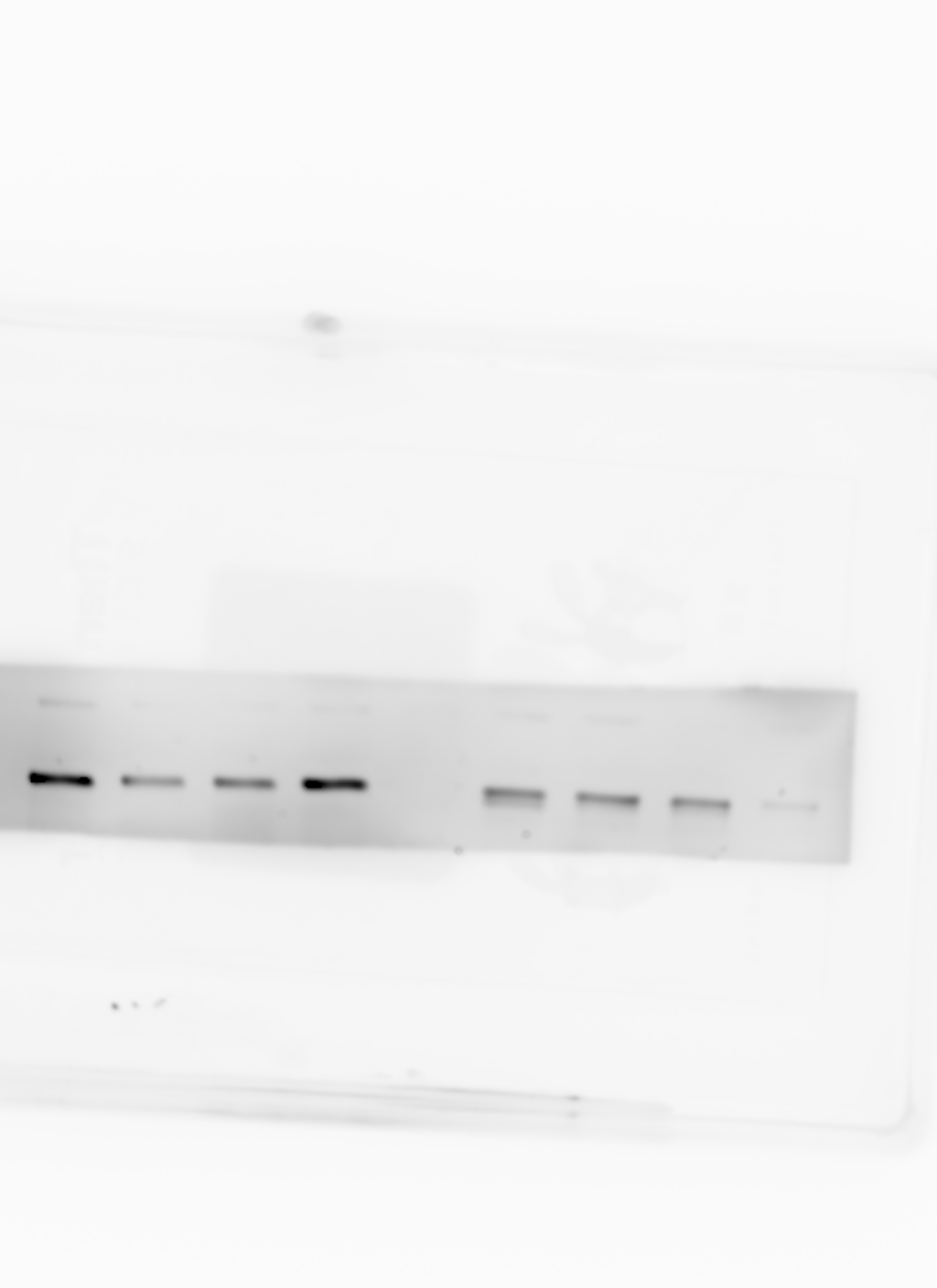

Supplement: Figure 2—source data 3. [file elife-88874-fig2-data3.zip › Fig 2D/iTreg NFATc2.tif]

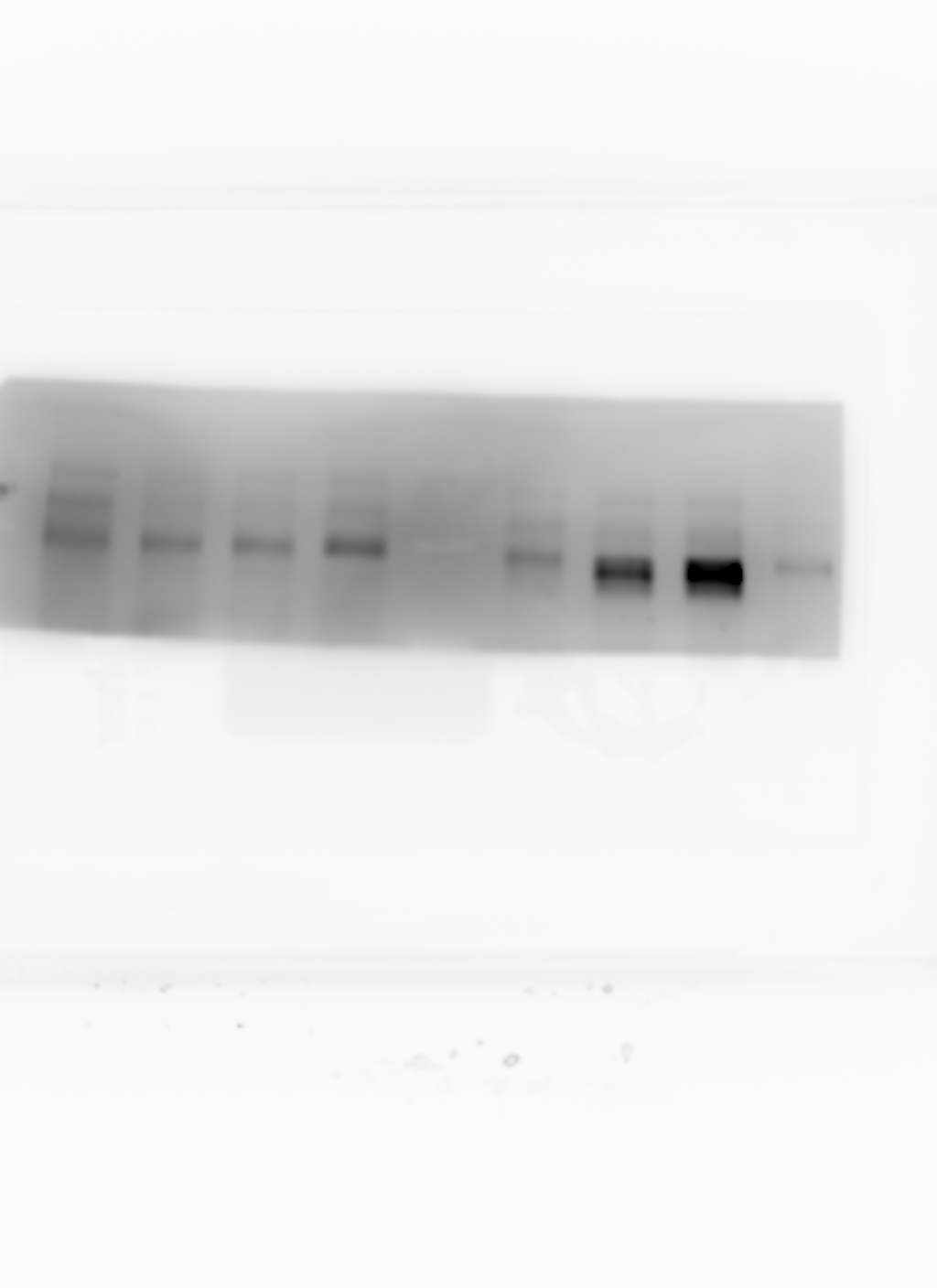

Supplement: Figure 2—source data 3. [file elife-88874-fig2-data3.zip › Fig 2D/iTreg NFATc1.tif]

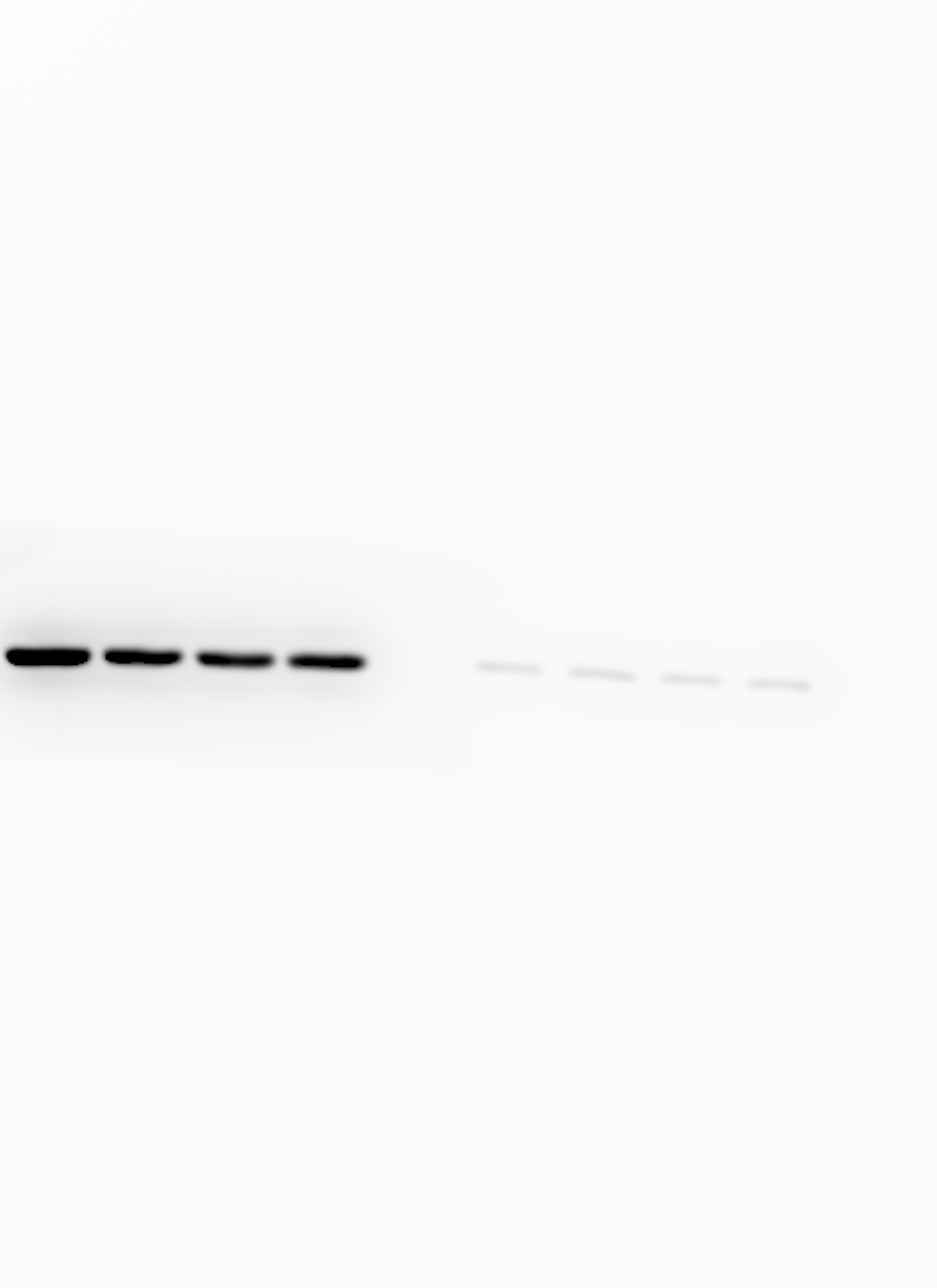

Supplement: Figure 2—source data 3. [file elife-88874-fig2-data3.zip › Fig 2D/iTreg Actin.tif]

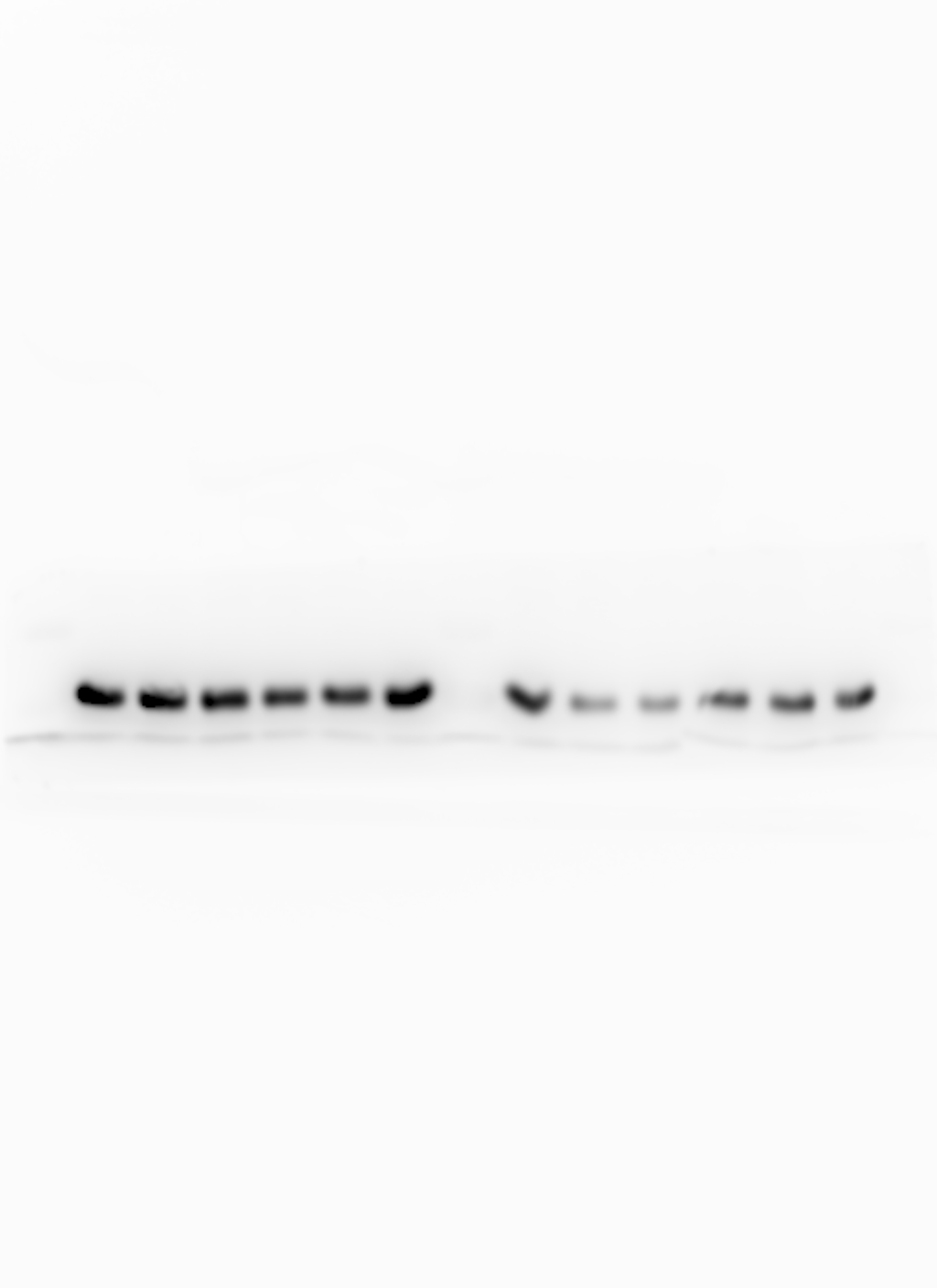

Supplement: Figure 2—source data 3. [file elife-88874-fig2-data3.zip › Fig 2D/Tconv&tTreg Gapdh.tif]

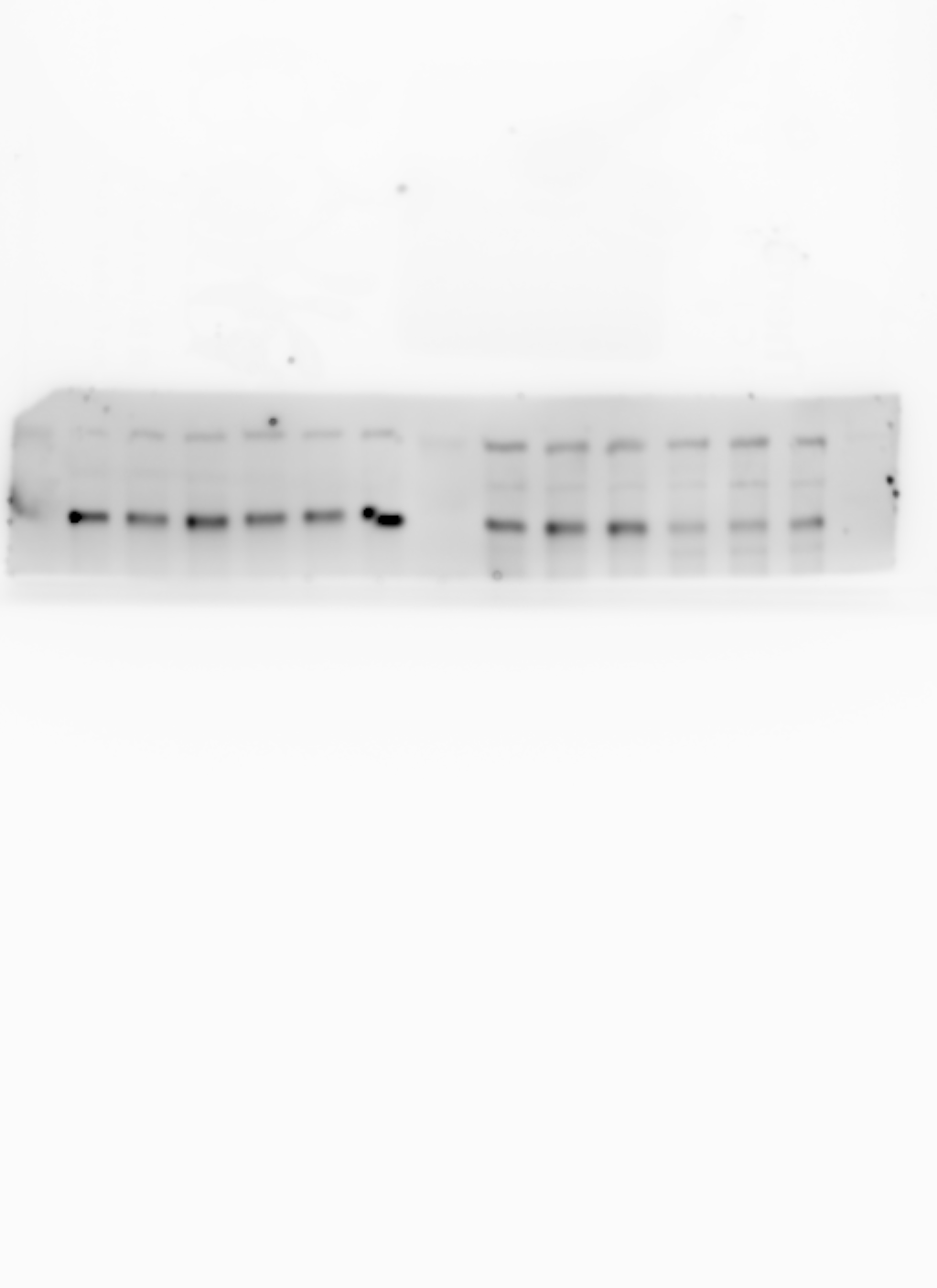

Supplement: Figure 2—source data 3. [file elife-88874-fig2-data3.zip › Fig 2D/Tconv&tTreg NFATc2.tif]

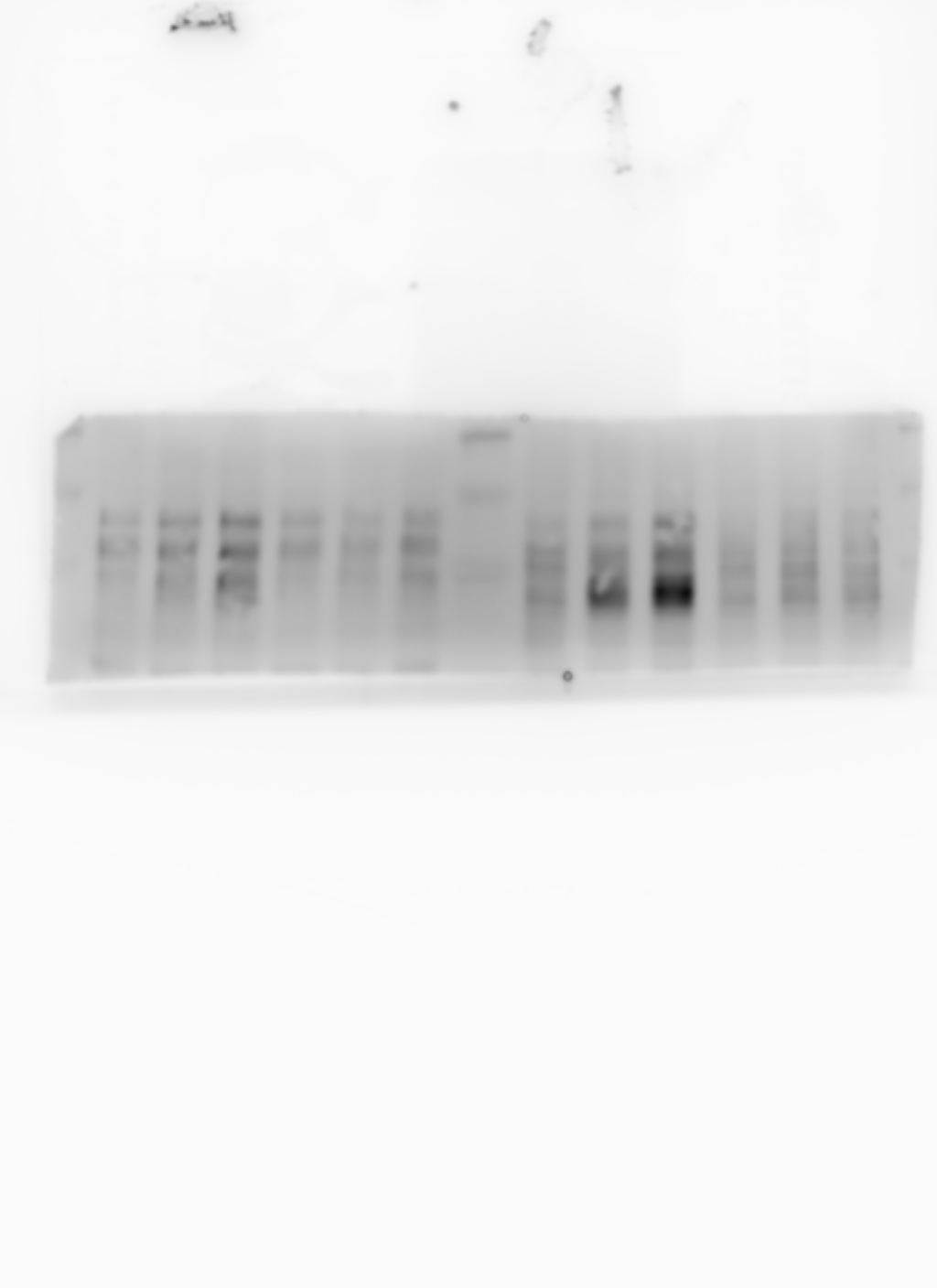

Supplement: Figure 2—source data 3. [file elife-88874-fig2-data3.zip › Fig 2D/Tconv&tTreg NFATc1.tif]
